# A Potent Antioxidant Endogenous Neurohormone Melatonin, Rescued MCAO by Attenuating Oxidative Stress-Associated Neuroinflammation

**DOI:** 10.3389/fphar.2020.01220

**Published:** 2020-08-21

**Authors:** Li Ling, Abdullah Alattar, Zhen Tan, Fawad Ali Shah, Tahir Ali, Reem Alshaman, Phil Ok Koh, Shupeng Li

**Affiliations:** ^1^ Department of Endocrinology, Shenzhen Nanshan People’s Hospital and the 6th Affiliated Hospital of Shenzhen University Health Science Center, Shenzhen, China; ^2^ Department of Pharmacology and Toxicology, Faculty of Pharmacy, University of Tabuk, Tabuk, Saudi Arabia; ^3^ Health Management Center, Shenzhen University General Hospital, Shenzhen University Clinical Medical Academy, Shenzhen University, Shenzhen, China; ^4^ Riphah Institute of Pharmaceutical Sciences, Riphah International University, Islamabad, Pakistan; ^5^ Department of Comparative Biology and Experimental Medicine, Faculty of Veterinary Medicine, Hotchkiss Brain Institute, Cumming School of Medicine, University of Calgary, Calgary, AB, Canada; ^6^ Department of Anatomy, College of Veterinary Medicine, Research Institute of Life Science, Gyeongsang National University, Jinju, South Korea; ^7^ State Key Laboratory of Oncogenomics, School of Chemical Biology and Biotechnology, Shenzhen Graduate School, Peking University, Shenzhen, China

**Keywords:** melatonin, middle cerebral artery occlusion, neuroinflammation, antioxidant, ischemic stroke

## Abstract

Ischemic stroke is an acute neurological syndrome either due to permanent or temporary obstruction of blood. Such obstruction immediately triggers abrupt pathological cascading processes, which collectively lead to neuronal cell death. Oxidative stress and neuroinflammation in ischemic stroke are critical regulating events that ultimately lead to neuronal death. Complicated interplay exists between the two processes which occur through several stages. Most often, oxidative stress precedes the inflammatory mechanisms and includes several interconnected cascades that underlie the ischemic stroke pathology. In continuation of the previously published data, here, we further ruled out the protective role of melatonin in focal cerebral ischemic injury model. Administration of 5 mg/kg dose of melatonin 30 min prior to ischemia reduced brain infarction associated with sequentially rescued neuronal apoptosis. Furthermore, melatonin attenuated neuroinflammatory markers and reactive oxygen species (ROS), induced by ischemic stroke, *via* halting the key players of mitogen stress family (p38/JNK). Besides, melatonin modulated the endogenously produced antioxidant enzyme, thioredoxin (Trx) pathway. These broader therapeutic efficacies of melatonin suggest that melatonin could be further investigated for its diverse therapeutic actions with multiple targets in recovering, preventing and halting the detrimental outcomes of MCAO, such as elevated oxidative stress, neuroinflammation, and neurodegeneration.

## Introduction

Globally, ischemic stroke is the most devastating human health condition and the major leading cause of impaired health and death in these modern technologically advanced and industrialized era (Feigin et al., 2014). Ischemic stroke results in severe physiological and neurological impairment (Boden-Albala et al., 2019). The pathophysiological complication of ischemic stroke depends largely on the area and type of occlusion, which can be either permanent or transient. Such occlusion can obstruct the supply of oxygenated blood to the brain. Furthermore, the supply of essential micro molecules is also affected by this impediment (Firan et al., 2020; Hui et al., 2020). The characteristic pathological attributes include loss of ionic gradient, as a result of reduced adenosine triphosphate (ATP) supply, which ultimately provokes membrane depolarization and calcium surge. Glutamate amassing further augments calcium release along with inflammatory and apoptotic mediators (Bruno et al., 2001). This metabolic crisis is followed by the prompt activation of several cytotoxic and pathological cascading events. The subsequent disturbances of cellular dysregulation further incorporate secondary tissue damage such as neuroinflammatory reactions. The inflammatory mediators have a significant role in stroke pathophysiology, due to the instant release of neuroinflammatory markers within the first hour of ischemic stroke (Tuttolomondo et al., 2012; Wang et al., 2013). Subsequent activation of glia cells further creates a vicious cycle of inflammatory cascades, in which release of inflammatory cytokines (tumor necrotic factor/TNF-α) from microglia further bolster the glia cell architecture towards inflammation. Furthermore, a secondary surge of proinflammatory cytokines such as interleukin-1 (IL-1β), and interleukin-6 (IL-6), contributes to a higher infarct volume in humans and thus further complicates the clinical prognosis. Antithrombotics are the first-line therapeutic agents against this life-threatening condition and include activase and tenecteplase (TNKase) (Melandri et al., 2009). The associated short therapeutic window, however, is largely considered as the potential limiting factor for these thrombolytic agents.

The impairment in the endogenous hormones has been implicated in several diseases such as cancer, cardiovascular, and age-associated neurodegenerative diseases. It has been widely reported that stroke mainly aﬀect the older population, which may be associated with a deficiency of endogenous hormones, such as estrogens, progesterone, and melatonin (Singh et al., 2008; Dodda et al., 2019). Among these the most promising is melatonin as the people become older, the melatonin level declined, which might be associated with the risk factor of ischemic stroke and make the people more vulnerable towards the stroke. A significant consideration is given to multiple synthetic compounds that mimic the endogenously produced hormones such as estrogens, progesterone, and melatonin due to their promising biological activities. Several studies reiterated the neuroprotective effects of melatonin, which is an indoleamine secreted mainly from the pineal gland, and other tissues (Messner et al., 2001; Stefulj et al., 2001; Venegas et al., 2012). Furthermore, the neuroprotective effects of melatonin in the ischemic brain model have been observed a long time ago, but still different studies suggest different molecular mechanisms for melatonin. Currently proposed mechanisms include including maintaining Ca2+ homeostasis (Onaolapo et al., 2019), suppressing inflammatory response (Azedi et al., 2019), decreasing oxidative stress, modulating stem cell survival (Watson et al., 2016), attenuating endoplasmic reticulum stress (Lin et al., 2018) and by modulation of microglia phenotypic responses (Liu et al., 2019). These beneficial effects of melatonin could be attributed mainly to its lipophilic nature that facilitates its rapid transportation to the brain (Yeleswaram et al., 1997). Second, the ubiquitous distribution of melatonin receptors in the brain leads to extensive interaction with some important intracellular proteins including nuclear receptor ROR/RZR, quinone reductase 2 (MT3), and calmodulin which participate in the regulation of circadian and seasonal rhythms (Benitez-King et al., 1993; Tan et al., 2007). ROR/RZR are nuclear receptor superfamily involved in the immunomodulatory and anti-tumor effects (Karasek et al., 2002; Winczyk et al., 2002). These effects are further supplemented by the large therapeutic window of melatonin, which makes this neurohormone an appropriate therapeutic option not only in brain disorders but also in other conditions. In this context, Ramos et al. demonstrated a detailed neuroprotective profile of melatonin in cerebral ischemia by targeting several signaling pathways, such as the pro-survival PI3K/Akt, mitogen-activated protein kinases (MAPKs), oxidative stress-related erythroid 2-related factor 2 (Nrf2), endothelin-1, and the N-methyl-D-aspartate receptor2A (NR2a)-dependent pathway (Shah et al., 2014; Shah et al., 2018b; Shah et al., 2019).

Current trends of drug development research have changed significantly due to repetitive failures of clinical trials. Several preclinical strategies have been demonstrated to inhibit delayed inflammatory response, but none of them were translated into effective clinical findings (Sughrue et al., 2004). Therefore, the underlying molecular and cellular mechanisms of oxidative stress-induced inflammation should be further delineated. Although several studies have investigated the favorable effects of melatonin against ischemic stroke, very few reports demonstrated the effect of melatonin on oxidative stress-induced inflammation and the role of the NF-κB/Trx pathway in an ischemic stroke model. Therefore, the current study was undertaken to evaluate the role of melatonin in cellular protection against oxidative stress-associated neuroinflammation in ischemic stroke.

## Materials and Methods

### Animal Housing, Grouping, and Drug Treatment

**Figure 1:**
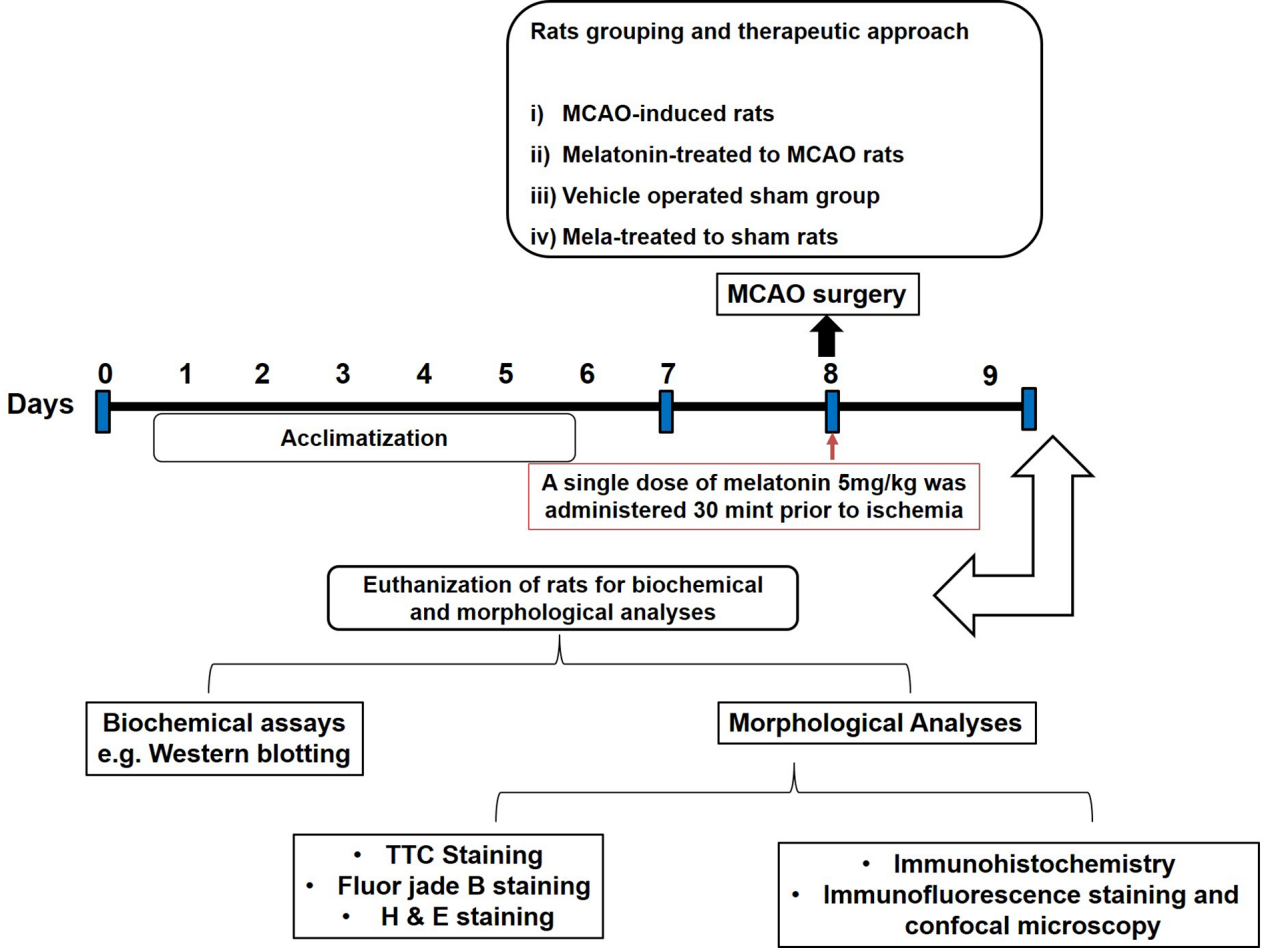
After 1 week of acclimatization, randomly selected male Sprague-Dawley were divided into the following groups: (1) Vehicle-treated sham rats; (2) MCAO rats; (3) Pre-melatonin administration to rats undergoing MCAO; (4) Pre-melatonin administration to sham rats. Melatonin or vehicle was administered as a single dose (5 mg/kg), all rats brain was isolated for further biochemical and immunohistochemical analyses.

Sprague-Dawley (SD) male rats weighing between 250–300 g and approximately 8-10 weeks old were acquired from an institutional breeding facility and were kept under a controlled environment, Peking University Shenzhen Graduate School. The animals were maintained in plastic cages, under equal light/dark period at room temperature with free access to food to facilitate experimental procedures and extra care was practiced to avoid unnecessary stressful events. All experimental procedures were pre-approved from the Institutional Animal Care and Use Committee of Peking University Shenzhen Graduate School (Approval number: AP0013002) and as such were strictly adhered to, in addition, to ARRIVE guidelines with few exceptions. We did not apply human endpoints for euthanizing the rats as the permanent MCAO model (24 h) is the most stressful invasive procedure and in which limited mobility with severe suffering is an established documented protocol, and by this our group was more interested in rats that survive this period. By this, we did not euthanize any rats until 24 h of the ischemic period. 4 groups of rats were designed, each with n=12 (**Figure 1**): 1. Sham group, rats in this group were administered saline as a vehicle; 2. MCAO group, rats in this group were exposed to invasive permanent middle cerebral artery occlusion; 3. Mela + MCAO group, rats in this group received melatonin prior to MCAO surgery; 4. Mela + Sham group, Melatonin was administered to sham rats.

We previously optimized the dosage regimen of melatonin (Sigma, St. Louis, MO, U.S.A) (Shah et al., 2014). Herein, we administered melatonin (dissolved in saline) or a vehicle at 5 mg/kg intraperitoneal dose prior to ischemia. A total of 7 animals have died, 4 from the ischemic group, 3 from melatonin treated groups, which we further adjusted by supplementing more animals. The reported reason for this mortality is edema formation, BBB leakage, and hypothalamic shutdown. The ethics committee is mostly aware of the mortality in experimental setup particularly in this model, as we constantly engaged for our work with this committee.

### MCAO Surgery

MCAO surgery was performed as per our previously published data (Sughrue et al., 2004; Shah et al., 2016; Shah et al., 2018a). Briefly, the animals were anesthetized by a cocktail of xylazine (9 mg/kg) and ketamine (91 mg/kg) and were placed in a heated cloth in a ventral position under a lamp. A central surgical incision was made, keeping the incision towards the right quadrant. The superficial muscle tissue was dissected out to probe for the right common carotid artery (CCA). Moreover, the whitish vagus nerve is carefully separated from the lining of CCA until the bifurcating point is reached. At this point, the two divergent branches of CCA, the external and internal carotid arteries (ECA and ICA) were identified and set free from surrounding tissues. Superior thyroid artery and the occipital artery which are small protrusions of ECA were identified and knotted with a thin black (6/0) silk (Sterile braided non-absorb SK-61, AILEE.CO, South Korea) and eventually ligated to allow free movement of the ECA. The ECA was then permanently tied with silk (6/0) near the hyoid bone located above the ligated superior thyroid artery. Throughout this procedure, extra care was exercised to avoid excessive bleeding. To induce the symptoms of ischemic stroke, the ECA was pierced, and subsequently, blue nylon silk (3/0, Sterile braided non-absorb NB319, AILEE.CO, South Korea) of 3 cm with a bulging tip, was introduced from the opening of the ECA and proceeds to the ICA and until finally to the opening of the middle cerebral artery (MCA). 24 h after occlusion, all animals were subjected to carbon dioxide (CO2) euthanasia. No insertion was made in the sham group, although the sham group was subjected to the same stressful setup. The only disadvantage in this method is the lack of Doppler effect and relative blood flow measurement, though we constantly do the occlusion with suitable experimental skills.

### Triphenyl Tetrazolium Chloride (TTC) Staining and Histological Preparation

All rats were sacrificed under the same condition of anesthesia, and brain tissue was collected. Three-millimeter thick coronal slices were cut starting from the frontal lobe or occipital lobe. The slices were stained by incubating in freshly prepared TTC solution (2% in PBS) in a water bath until a demarcation of white-red was noticed. The slices were fixed in 4% paraformaldehyde solution and photographed for percent infarct area determination (Shah et al., 2019). ImageJ program was used for infarct area determination and presented as percent total area. To compensate for the false reading of edema, the following formula was applied:

Corrected infarct area = left hemisphere area of the brain − (hemisphere area from the right brain − the area of infarction)

These sections were then processed for paraffin blocks preparation using an embedding machine and rotary microtome n=6/group.

### Haematoxylin Eosin (H& E) Staining

Tissue slides were subjected to deparaffinization protocol, which started from three different xylene treatments for 10 min and then rehydrated in graded alcohol preparation (commencing from 100% to 70%, each wash for 5-10 min). These slides were subsequently rinsed with distilled water to clear ethanol remaining and immersed in hematoxylin for 10 min at room temperature. After due time, the slides were washed with distilled water and observed under the microscope to ensure nuclear staining, otherwise, incubation time with hematoxylin was increased, followed by 1% HCl solution treatment for a short interval, rinsed with distilled water and immediately treated with 1% ammonia water, followed by rinsing with water. The slides were then stained with pinkish eosin solution for the appropriate time and were then rinsed in water and kept under room temperature for air-drying. This step was followed by gradient ethanolic dehydration and fixation in absolute xylene (reverse deparaffinization protocol) and mounted with a glass coverslip. By using an Olympus light microscope at 40x magnification scale, slides were analyzed for the extent of neuronal death and survival using an ImageJ program.

### Immunohistochemical Staining (IHC) and Microscopic Analysis

IHC was achieved according to our previously published report (Sung et al., 2012). Briefly, after deparaffinization protocol as demonstrated above, slides were processed for antigen retrieval with proteinase K and washed with 0.1 M PBS. The peroxidase activity was quenched by applying a diluted hydrogen peroxide solution (3% in methanol). After washing with 0.1 M PBS, slides were treated with normal goat serum (NGS). Slides were then incubated for a whole night with primary antibodies such as mouse anti-phosphorylated-c-Jun N-terminal kinase (p-JNK), rabbit anti-interleukin (IL-1β), mouse anti-tumor necrotic factor (TNF-α) and rabbit anti-p-NF-κB, (Santa Cruz Biotechnology, Inc) at 1:100 dilution. The next morning, slides were incubated subsequently with biotinylated tagged secondary antibody (dilution 1:50), with ABC Elite kit (Santa Cruz Biotechnology) and stained in DAB solution. This step was followed by gradient ethanolic dehydration and fixation in absolute xylene (reverse deparaffinization protocol) and mounted with a glass coverslip. By using an Olympus light microscope at 40x magnification scale, slides were analyzed for hyperactivated p-JNK, IL-1β, p-NF-κB, and TNF-α using an ImageJ program.

### Immunofluorescence Staining and Confocal Microscopy

Same deparaffinization and antigen retrieval protocol were adopted, followed by PBS rinsing. Immuno-slides were incubated with 5% normal serum depending upon secondary antibody employed, followed by whole night incubation at 40C with primary antibodies. The antibodies used were, glial fibrillary acidic protein (GFAP), cyclooxygenase-2 (COX-2), nitric oxide synthase-2 (NOS-2), p-NF-κB, and thioredoxin (Trx) from (Santa Cruz Biotechnology, Inc) with dilution factor 1:100. The following morning, slides were treated with secondary antibodies that were tagged with a fluorescent dye (Santa Cruz Biotechnology) for 1.5 h avoiding the light source. Commercially available Ultra Cruz mounting max (consisting of DAPI and mounting media) were used for coverslipping and were photoshoot by a confocal microscope (Flouview FV 1000 Japan) with appropriate scale and analyzed by ImageJ software.

### Fluoro-Jade B (FJB) Staining

After deparaffinization, slides were immersed in 1% NaOH, followed by diluted graded ethanolic solution (starting from 80% to 70%), subsequently with distilled water and were transferred to Coplin jar containing 0.06% KMNO4, rinsed with distilled water and immediately treated with 0.1% acetic acid solution and 0.01% FJB. After rinsing, slides were mounted using DPX mounting medium and then photoshoot by confocal microscopy and quantified by Image. Although, the exact mechanism by which FJB binds to degenerative neurons is not well understood. Schmued et al. stated that FJB, an anionic derivative of fluorescein, possibly interacts with intracellular polyamines and thereby stains degenerative neurons (Schmued and Hopkins, 2000). Furthermore, FJB has an advantage over its predecessor Fluoro-Jade, due to its greater specific affinity for degenerating neurons.

### Western Blot Analysis

For relative protein expression, we followed the same methodological protocols of western blot as our previously published data (Gim et al., 2013). The whole tissue extract was centrifuged and resulted supernatant was carefully isolated. Bicinchoninic acid (BCA) kit (Pierce, Rockford, IL, USA) was utilized to determine protein concentration according to the guidelines provided by the manufacturer. 30 µg per sample of protein were electrophoresed and were transferred to polyvinylidene fluoride (PVDF) membranes (Millipore, Billerica, MA, USA). After blocking, the membrane PVDF was incubated with primary antibodies for the whole night at the refrigerator. The next morning secondary antibodies were applied, and the ECL detection reagent was used for x-ray bands. The antibodies used include anti-TNF-α, anti-p-JNK, Anti-JNK, anti-COX2, anti-IL-1β, anti-NOS2, anti-nuclear factor erythroid 2-related factor 2 (Nrf2), anti-p-NF-κB, anti-heme oxygenase-1 (HO-1), anti-Trx, and anti-β-actin (Santa Cruz, Biotechnology, CA, USA). The anti-mitogen activated protein kinase phosphorylated-P38 (MAPKp-P38) and anti-total-P38 were purchased from (Cell Signaling Technology, CST), and were used at dilutions 1:1000.

### Data and Statistical Analysis

All the data are shown as means ± standard error of the mean (SEM) and were analyzed either by one-way or two-way ANOVA followed by Bonferroni Multiple Comparison as post-hoc *via* prism-7. ImageJ software was used for the analysis of protein bands and morphological data.

## Results

### Pre-Treatment Effect of Melatonin on Brain Infarction and Neuronal Cell Loss

**Figure 2:**
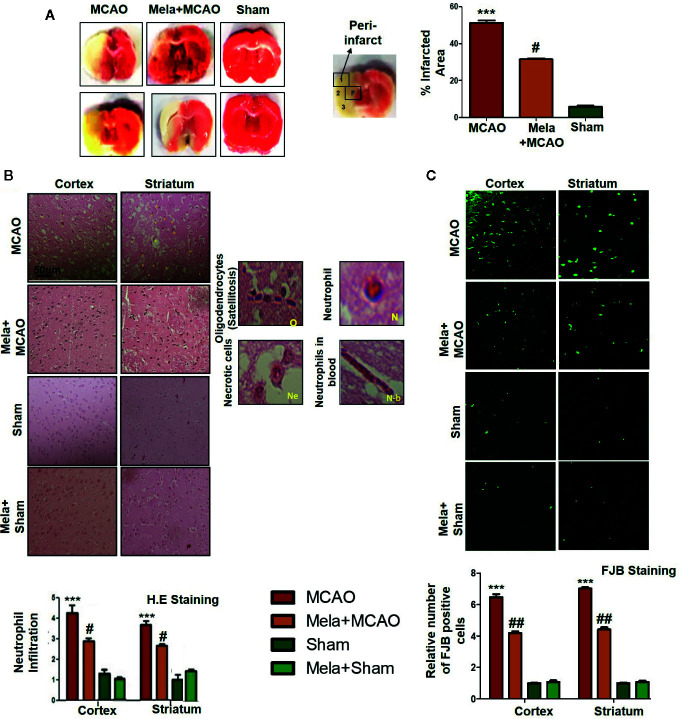
**(A)** TTC staining was done to demarcate between ischemic and non-ischemic areas and to evaluate the neuroprotective effects of melatonin (n=5/group). The regions of interest (ROIs) selected were indicated by 1 and 4, respectively showing the frontal cortex and striatum. The parietal cortex is shown by 2, and the piriform cortex by 3. TTC staining was analyzed by one ANOVA followed by a non-parametric test (as Kruskal-Wallis test). Significance ^∗∗∗^p < 0.001 showing significant difference relative to vehicle operated sham group, and ^#^p < 0.05 showing significant difference relative to MCAO group **(B)** Representative images of H&E staining showing the degree of neutrophil infiltration in cortical and striatal tissue after ischemic insult, scale bar = 50 μm, magnification 40× and (n = 5/group). These tissue slides were processed from the stained TTC coronal sections after fixation in 4% paraformaldehyde. Infiltrated oligodendrocytes (O), neutrophils (N), and neutrophils in blood (N.b) are shown. Necrotic cells (Ne) with scalloped appearance are shown. Data were analyzed by two ways ANOVA followed by post-hoc Bonferroni Multiple Comparison test using graph-pad prism-5 software **(C)** Representative images of FJB staining, scale bar = 30 μm. These tissue slides were processed from the stained TTC coronal sections after fixation in 4% paraformaldehyde, and the analyzed area is frontal cortex and striatum.). Symbol ∗∗∗ showing significant difference relative to vehicle operated sham group and the value is p < 0.001 or p < 0.01, respectively, while # or ## showing significant difference values of p < 0.05 or p < 0.01, respectively, relative to MCAO group. TTC, 2,3,5-Triphenyltetrazolium chloride; FJB, Fluoro-Jade B; H&E, hemotoxylin and eosin.

Ischemic brain injury produces a significant distortion of a neuronal cell in the core and penumbral region, which includes striatum and frontal cortex respectively. TTC staining was used to distinguish between intact and infarcted tissue (**Figure 2A**, n=6/group). Melatonin significantly reduced infarction size induced by MCAO (p<0.05, **Figure 2A**). The neuronal damage in ischemic injury could be due to necrosis in the infarcted core due to severe depletion of blood flow and ATP supply or due to apoptosis in the penumbral region that surrounds the core. Depending on brain location, broad crosstalk exists between necrotic and apoptotic cell death, due to analogous triggering agents such as glutamate and ROS. We, therefore, selected the peri-infarct regions as indicated by number 1 for the frontal cortex and number 4 for the striatum, as regions of interest (ROIs) (**Figure 2A**) for all further morphological analysis.

The H & E staining revealed the extent of neuronal injury provoked in the frontal cortex and the striatum. The neuropil of the ischemic cortex exhibited considerable aberrant morphological features relative to the sham group. However, melatonin treatment attenuated this damage to a significant extent (**Figure 2B**, p<0.05). The characteristic alteration observed in the ipsilateral cortex and striatum included scalloped neuronal structure accompanied by slight changes in color staining, vacuole formation, and neutrophil infiltration (**Figure 2B**). Moreover, FJB staining was performed to observe the anti-apoptotic effect of melatonin, which demonstrated a relative attenuated level of FJB-positive cells in (**Figure 2C**, p<0.01) relative to MCAO (**Figure 2C**, p<0.001).

### Pre-Treatment Effect of Melatonin on Outcomes of MCAO-Mediated Stress-Associated MAPK p-P38/p-JNK Pathways

**Figure 3:**
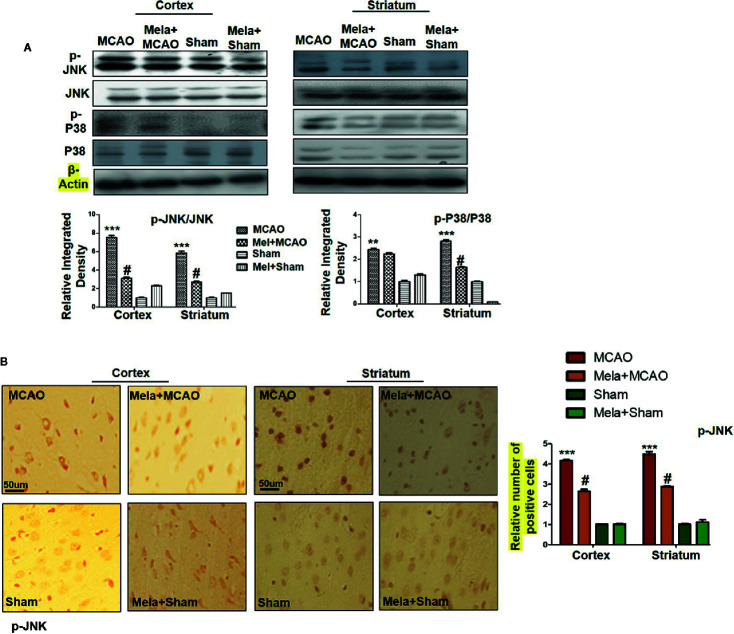
**(A)** The immunoblot results of p-P38, total P-38, p-JNK, and total JNK from the ipsilateral cortex and striatum following MCAO. The immunoblot bands were quantified using ImageJ software, and the statistical differences are indicated in the corresponding graphs. The data are expressed as the mean ± SEM for n = 6 rats/group, and the number of experiments = 3. β-Actin was used as a control. Data were analyzed by two-way ANOVA followed by post-hoc Bonferroni Multiple Comparison test using graph-pad prism-5 software. The brain tissue for Western blot was collected after 24 h of vehicle or melatonin treatment and stored at −80°C until used. Symbol ∗∗∗ or ∗∗ showing significant difference relative to vehicle operated sham group and their values are p < 0.001 or p < 0.01 respectively, while # showing significant difference values of p < 0.05 relative to MCAO group. **(B)** The presented images indicated Immunoreactivity of p-JNK in the cortical and striatum tissue of rat brain. The p-JNK exhibits cytoplasmic localization. The data are expressed as the mean ± SEM for n = 5 rats/group, and the number of experiments = 3. Scale bar = 50 μm, magnification 40×. The immunohistochemistry slides were processed from the stained TTC coronal sections after fixation in 4% paraformaldehyde. From the thick coronal TTC sections, paraffin blocks were made, and later 4-μm-thin coronal sections were prepared by a rotary microtome. The symbol ∗∗∗ showing significant difference relative to the vehicle operated sham group and the value is p < 0.00, while # showing significant difference value of p<0.05 relative to the MCAO group. p-JNK, phospho c-Jun N-terminal kinase; JNK, c-Jun N-terminal kinase; p-P38, phospho-P38.

The MAPK-p38/p-JNK pathway is predominantly activated due to oxidative stress. Besides, inflammatory mediators such as p-NF-κB and Toll-like receptor ligands could also activate p-JNK which suggested a close association of MAPK p-p38/p-JNK pathway in inflammation (Tu et al., 2010; Yao et al., 2013). Moreover, previous reports reiterated the pro-apoptotic role of MAPK such as p-JNK and p-p38. To examine the neuroprotective effects of melatonin on P38/JNK expression, we did Western blotting, and the results demonstrated higher p-JNK and p-P38 expression following permanent MCAO injury in comparison to sham. The pre-treatment dosage regimen of melatonin attenuated MCAO-mediated stress associated MAPK p-P38/p-JNK pathways (**Figure 3A**). Moreover, immunohistochemical results validated our Western findings (**Figure 3B**).

### Pre-Treatment Effect of Melatonin on Endogenous Antioxidant Nrf2/HO-1/Trx Pathways

**Figure 4:**
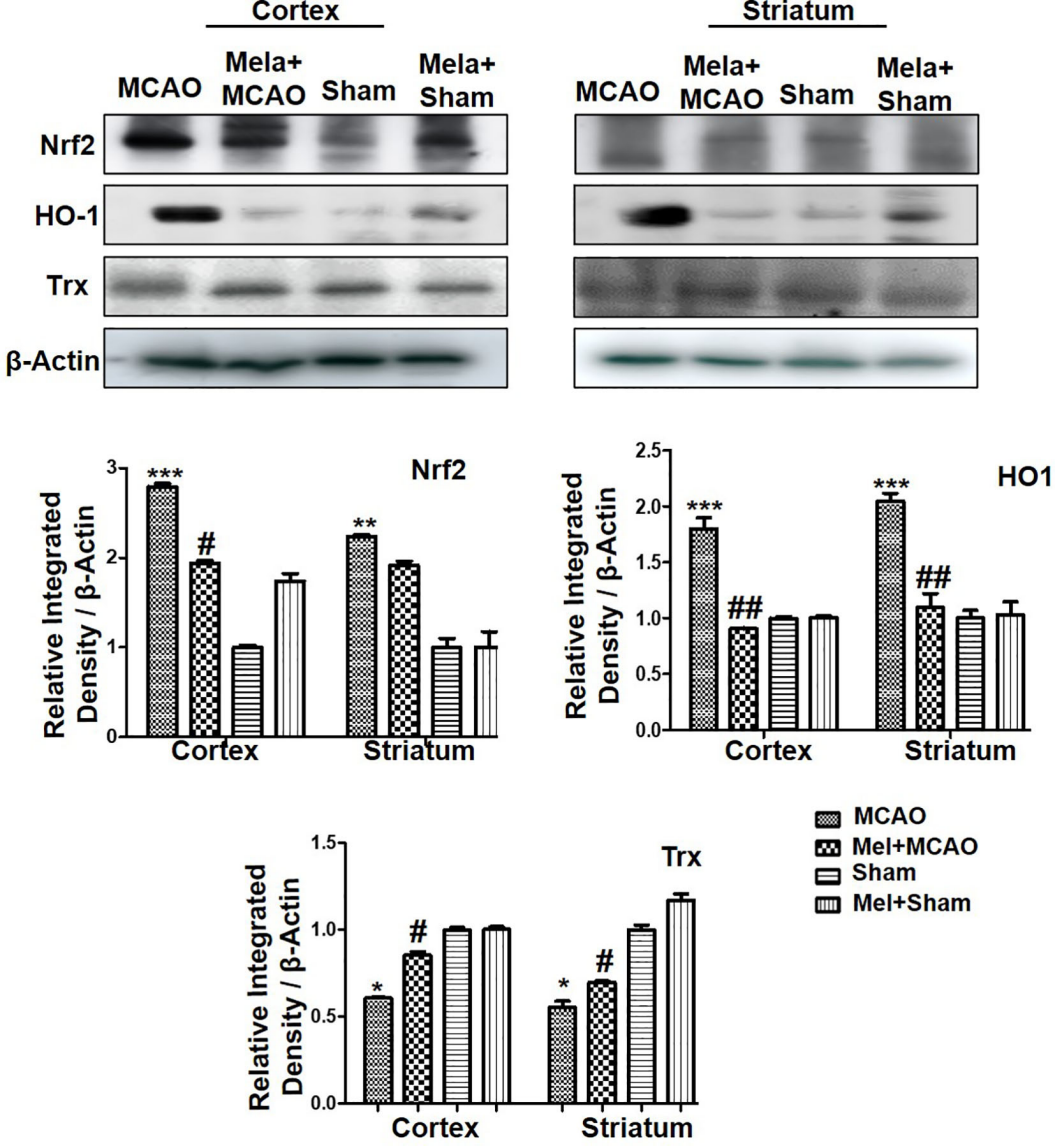
The immunoblot results of Nrf2, HO-1, and Trx in the cortical and striatum tissue of rat ipsilateral brain. The immunoblot bands were quantified using ImageJ software, and the statistical differences are indicated in the relative graphs. The data are expressed as the mean ± SEM for n = 6 rats/group, and the number of experiments = 3. Data were analyzed by two-way ANOVA followed by post-hoc Bonferroni Multiple Comparison test using graph-pad prism-5 software β-Actin was taken as a loading control. Significance = ^∗∗∗^p<0.00, ^∗∗^ or ^##^p<0.01, and ^#^ or ^∗^p<0.05. The brain tissue for Western blot was collected after 24 h of vehicle or melatonin treatment and stored at −80°C until used, Nrf2, nuclear factor erythroid 2-related factor 2; HO-1, heme oxygenase-1; Trx, thioredoxin.

Nrf2 acts as a master cellular homeostasis and stress response element in many neurodegenerative diseases. Nrf2 is an established endogenous antioxidant protein, that executes important protective effects by modulating the expression of several downstream antioxidant proteins. The inactive Nrf2 is localized to cytoplasm in a complex with Keap1, and upon dissociation of the dimer, Nrf2 translocates to the nucleus and activates potential endogenous antioxidant including downstream HO-1, superoxide dismutase (SOD) and glutathione (GSH) (Itoh et al., 2004; Kobayashi and Yamamoto, 2005; Vasconcelos et al., 2019; Yuan et al., 2020). Herein, we were interested in analyzing the effect of melatonin on Nrf2 and its downstream target HO-1. Furthermore, the effect of melatonin on Trx, a key member of the thioredoxin-peroxiredoxin family was also evaluated in the ischemic brain. The western blot images and corresponding histograms showed a relatively higher expression of Nrf2 and HO-1 24 h of permanent MCAO in comparison to the sham group (**Figure 4**). Interestingly, Trx was downregulated in the ischemic brain, while melatonin treatment significantly restored the expression level of Trx. Noticeably, we did not observe any effect on Nrf2 level (90-120 kDa) in the melatonin-treated groups (**Figure 4**).

### The Pre-Treatment Dosage Regimen of Melatonin Downregulates Ischemia Stress-Associated Neuroinflammation

**Figure 5:**
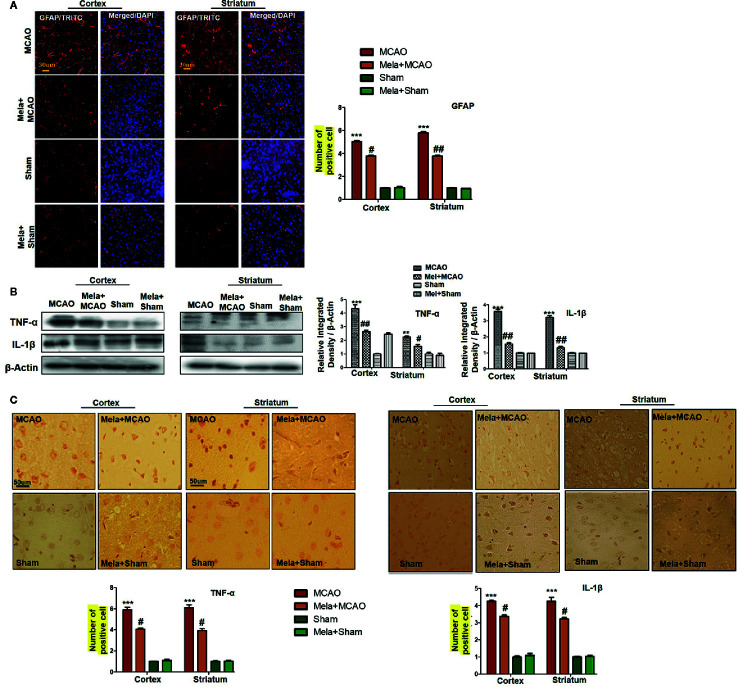
**(A)** Immunoreactivity of GFAP-positive cells in different groups are shown with magnification 40× and scale bar = 30 μm. The data are expressed as the mean ± SEM for n = 5 rats/group, and the number of experiment s= 3. The immunohistochemistry slides were processed from the stained TTC coronal sections after fixation in 4% paraformaldehyde. From the thick coronal TTC sections, paraffin blocks were made, and later 4-μm-thin coronal sections were prepared by a rotary microtome. The symbol ∗∗∗ showing significant difference relative to the vehicle operated sham group and the value is p<0.00, while # or ## showing significant difference value of *p* < 0.05 and *p* < 0.01, respectively, relative to MCAO group. **(B)** Effect of melatonin on inflammatory cytokines. Western blot results of TNF-α and IL-1β from the ipsilateral brain were analyzed by ImageJ. The statistical differences are indicated in the relative graphs. The data are expressed as the mean ± SEM for n = 6 rats/group, and the number of experiments= 3. β-Actin was used as a control. Data were analyzed by two-way ANOVA followed by post-hoc Bonferroni Multiple Comparison test using graph-pad prism-5 software. The brain tissue for western blot was collected after 24 h of vehicle or melatonin treatment and stored at −80°C until used. The symbol ^∗∗∗^ or ^∗∗^ showing significant difference relative to the vehicle operated sham group and their values are *p* < 0.00 or *p* < 0.01, respectively, while # or ## showing significant difference value of p<0.05 and p<0.01 respectively relative to MCAO group. Symbol ^∗∗^ showing significant difference relative to vehicle operated sham group and the value is p < 0.01. **(C)** Immunohistochemistry results for TNF-α and IL-1β are shown; scale bar = 50 µm; magnification, 40×. Tissue sections show correspondingly elevated expression of TNF-α and IL-1β after 24 h of permanent ischemia and both proteins show cytoplasmic localization. Significance = ^∗∗∗^p<0.001 and ^#^p<0.05. All the morphological data are expressed as the mean ± SEM for n = 5 rats/group. The immunohistochemistry slides were processed from the stained TTC coronal sections after fixation in 4% paraformaldehyde. The TTC sections were subjected to embedding and paraffin blocks were made and later 4-μm coronal sections were made by a rotary microtome. GFAP, glial fibrillary acidic protein; TNF-α, tumor necrosis factor; IL-1β, interleukin.

Resident immune cells such as glial cells play an integral role in the mediation of inflammation. Moreover, the glial cells particularly the microglia cells are involved both to release the cytokines and to release some trophic substances such as brain-derived neurotrophic factor-BDNF (Yu and Lau, 2000). We demonstrated the astrocytes activation by GFAP-reactive cells as astrocytes assume a characteristic appearance which we revealed by immunofluorescence. Likely, we noticed the increase in the pro-inflammatory cytokines (TNF-α and IL-1β), similar to previously reported observations (Davies et al., 1999). Melatonin treatment, on the other hand, attenuated these effects (**Figures 5A, B**), which was further validated by immunohistochemistry findings (**Figure 5C**).

### Effect of Pre-Treatment Dosage Regimen Melatonin on Outcomes of MCAO-induced Inflammatory Mediators

**Figure 6:**
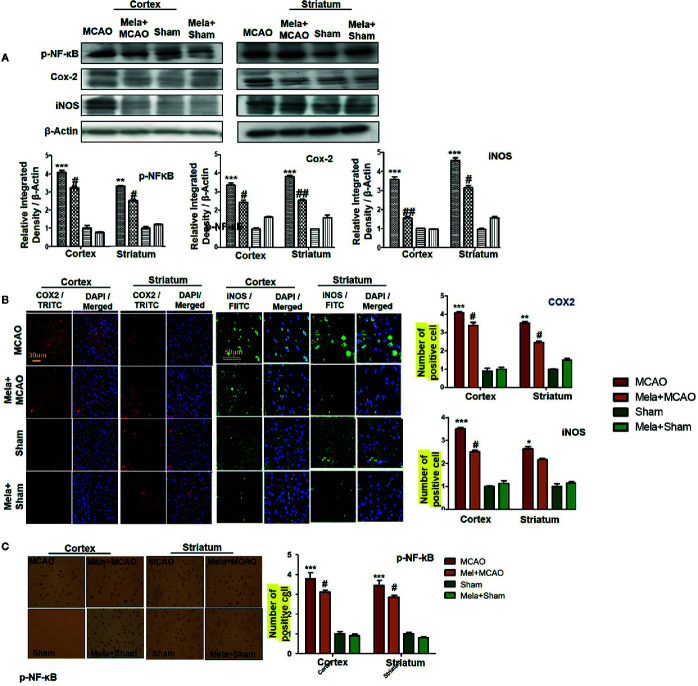
Melatonin downregulated the NF-κB signaling pathway. **(A)** The immunoblot results of p-NF-κB, COX2, and iNOS in the cortical and striatum tissue of rat ipsilateral brain. The immunoblot bands were quantified using ImageJ software, and the statistical differences are indicated in the relative graphs. The data are expressed as the mean ± SEM for n=6 rats/group, and the number of experiments = 3. β-Actin was used as a control. Data were analyzed by two-way ANOVA followed by *post hoc* Bonferroni Multiple Comparison test using graph-pad prism-5 software. The brain tissue for western blot was collected after 24 h of vehicle or melatonin treatment and stored at −80°C until used. The symbol ∗∗∗ showing significant difference relative to the vehicle operated sham group and the value is p < 0.001, while # or ## showing significant difference value of p < 0.05 and p < 0.01 respectively relative to MCAO group. **(B)** Immunofluorescence reactivity of COX2 and iNOS; scale bar = 30 and 50 µm; magnification, 40×. The COX2 and iNOS-positive cells were visualized by TRITC and FITC respectively and showed cytoplasmic localization. The data are expressed as the mean ± SEM for n = 5 rats/group and the number of experiments performed=3. Symbol ** or * showing significant difference relative to vehicle operated sham group and the values are respectively p < 0.01 and p < 0.05. **(C)** The presented images indicated Immunoreactivity of p-NF-κB in the cortical and striatum tissue of rat brain. The p-NF-κB exhibits nuclear localization, and the number of experiments= 3.; scale bar = 20. Significance = ***p < 0.001 ^##^p < 0.01. NF-κb, nuclear factor kappa light chain enhancer of activated B cells; COX2, cyclooxygenase; iNOS, inducible nitric oxide or nitric oxide synthase. Symbol ^∗∗^ or * showing significant difference relative to vehicle operated sham group and the values are respectively p < 0.01 and p < 0.05.

The binding of proinflammatory factors TNF-α and IL-1β to respective receptors triggers the sequential activation of downstream molecules such as ASK1, SEK1, and JNK. Collectively, this leads to proteasomal dependent IκB dissociation, and nuclear translocation of NF-κB to induce inflammatory transcription types of machinery like NOS2 and COX2 (Popp et al., 2009). These proteins were hyper expressed in western blot finding in the ischemic brain (p<0.001, **Figure 6A**). The melatonin pre-treatment dosage regimen significantly alleviated this level (p<0.05). Immunostaining was further performed to validate these findings for COX2, NOS2, and p-NF-κB (**Figures 6B, C**).

### Effect of Pre-Treatment Dosage Regimen of Melatonin on the p-NF-κB/Trx Pathways

**Figure 7:**
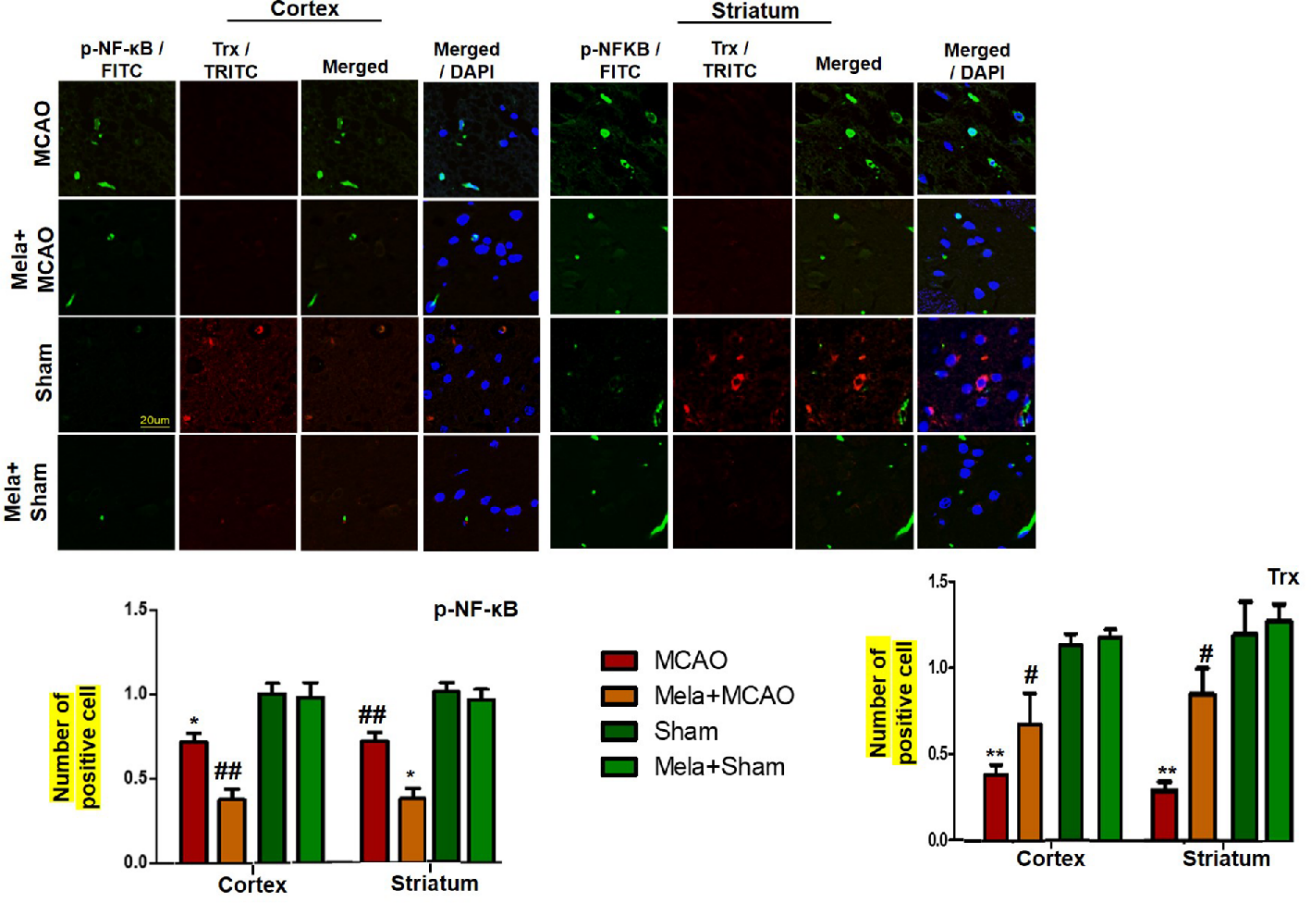
Effect of melatonin (5 mg/kg) on the p-NF-κB/Trx pathways **(A, B)** p-NFκB and Trx were colocalized by double immunofluorescence. Scale bar = 20 μm and (n=5/group). p-NFκB showed higher expression level accompanied with Trx down expression in the MCAO group. p-NF-κB and Trx were visualized by FITC and TRITC, respectively. Significance = * and ** shows significant difference relative to sham operated vehicle group and its value are p < 0.05, and p < 0.01. ^##^ shows significant difference relative to MCAO operated group and its value is p < 0.01. Significance = ^#^ or *p < 0.05; and ** or ^##^p < 0.01. FITC, fluorescein isothiocyanate; TRITC, tetramethylrhodamine.

To demonstrate the effect of melatonin on the co-association of p-NF-κB and Trx, we colocalized these proteins by double immunofluorescence (**Figure 7**). As demonstrated, elevated expression of p-NF-κB was observed accompanied by decreased Trx expression. Treatment with melatonin reversed the injury-induced reduction in the level of Trx.

## Discussion

Here, we demonstrated that the endogenous neurohormone melatonin attenuates the detrimental outcomes of MCAO-induced ischemic stroke, and the current study is the continuation of our previous studies to further extend the role of melatonin. We previously have shown that melatonin treatment affects the glutamate N-methyl-D-aspartate (NMDA) and alpha-amino-3-hydroxy-5-methyl-4-isoxazole propionic acid (AMPA) receptor signaling in cerebral cortex and striatum 24 h after permanent middle cerebral artery occlusion (MCAO) and attenuated ischemia-induced down-regulation of NMDA receptor 2 (NR2a), postsynaptic density-95 (PSD95) and increases NR2a/PSD95 complex association, which further activates the pro-survival PI3K/Akt/GSK3β pathway with mitigated collapsin response mediator protein 2 (CRMP2) phosphorylation (Shah et al., 2019). Moreover, we have also demonstrated that melatonin may act in a multimechanistic way by modulating the expression of several proteins in the ischemic striatum using a proteomic approach (Shah et al., 2018b). The degree and severity of neuronal injury induced by ischemia largely depend on the occlusion period. The infarct area in the permanent MCAO model frequently involves regions from neocortex and striatum (Lo et al., 2003). Furthermore, necrotic cell death primarily occurs at striatal tissue suggesting a dramatic decrease of blood to this whitish tissue (Fluri et al., 2015).

**Figure 8:**
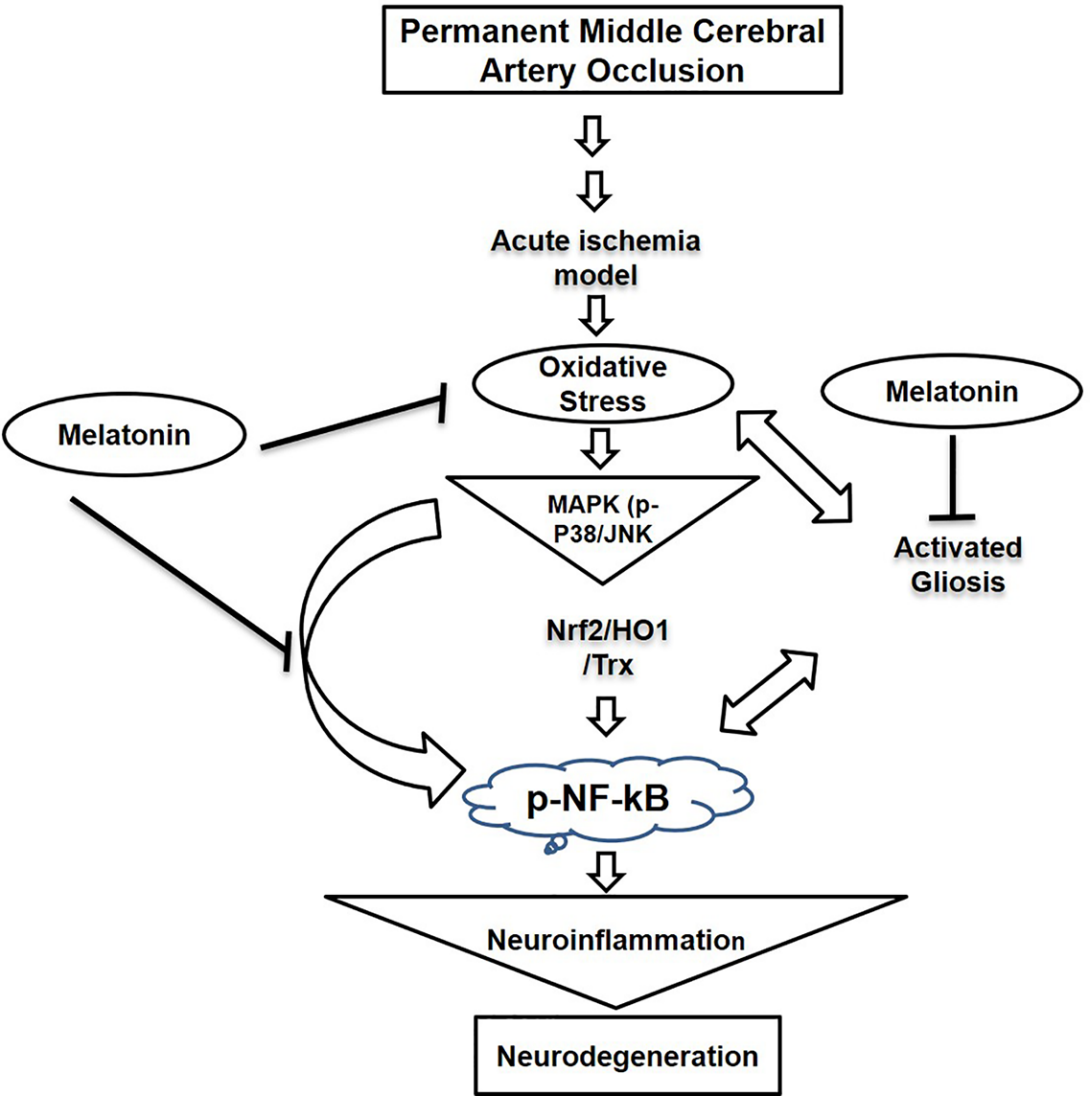
A graphical representation of the underlying neuroprotective mechanisms of melatonin against the MCAO-induced oxidative stress-mediated neuroinflammation and neurodegeneration.

Our results demonstrated that melatonin mitigated MCAO-mediated neuroinflammation and neurodegeneration *via* inhibiting apoptotic cell death, which could be mediated by mitigating inflammation and oxidative stress along with enhanced antioxidant capacity. Furthermore, melatonin had strong pleiotropic effects, both by alleviating ROS mediated neuroinflammation but also through the strengthening of intrinsic antioxidative mechanisms, such as Trx (**Figure 8**). Many studies reiterated that targeting different aspects of stroke injury such as oxidative stress and inflammation could be of specific therapeutic value. Therefore, more experimental studies are required to unveil such mechanisms (Kacimi et al., 2011).

Consistent studies showed the role of the JNK pathway in mediating inflammatory events. Furthermore, attenuating p-NF-κB expression could also reduce the infarct area by mitigating ROS generation through COX2 and iNOS. JNK and p38 both are implicated in apoptotic cell death by a cross-talk with inflammatory mediators such as elevated p-NF-κB expression. We speculate that melatonin can diminish neuronal susceptibility by negatively affecting mitogen kinase expression, which is further linked to cytokines expression. Inflammation significantly affects the therapeutic outcome of stroke by compromising clinical prognosis. Stroke induces translocation of resident and peripheral cells to the site of injury to contribute to inflammatory pathogenesis (Wang et al., 2013). These cells further release inflammatory mediators to exacerbate neuronal injury by several mechanisms.

Stress-related MAP Kinases including JNK and p38 are activated by TLR-4 agonists located on glial cells (Munoz et al., 2007), which in turn leads to mitochondrial apoptotic activation. Moreover, inflammatory cytokines are released from hypertrophic glial cells such as microglia and astrocytes instantly after the ischemic attack (Rothwell, 1991; Putcha et al., 2003; Caso et al., 2007; Wang et al., 2012). This process is further complicated by activated nuclear transcriptional machinery like NF-κB, which triggers the downstream inflammatory mediators COX-2 and INOS and thus aggravates the damage.

Furthermore, activation of NF-κB by TLR-4 further activates downstream toxic mediators like iNOS/NOS2 and COX-2 (Popp et al., 2009). It has been reported that the activity of COX-2 and NOS2 can be attenuated by inhibiting the TLR-4 receptor on glial cells (Ramos et al., 2017). Results from several other ischemic models suggested the anti-inflammatory potential of melatonin by downregulating the expression of NF-κB, COX-2, and iNOS (Luo et al., 2019). In our study, we examined the expression level of thiol protein Trx, modulates hemostatic redox reactions. Melatonin pre-treatment significantly enhanced the expression level of Trx, further supports the antioxidant notion of melatonin in ischemic injury. Moreover, how melatonin attenuates ischemic induced neuronal toxicities are yet to be explored, though the anti-oxidant nature of melatonin could be ascribed for these effects (Yeligar et al., 2010; Patiño et al., 2016; Guo et al., 2018).

Earlier studies reported the cross-talk of oxidative stress-induced inflammation process (Ali and Kim, 2015; Ali et al., 2018). Thus, therapeutic approaches should aim at regulating and subsiding the neuroinflammation as well as the accumulation of oxidative stress *via* stimulating the antioxidant enzymes. Melatonin acts as an endogenous potent antioxidant, free radical scavenger, and a regulator of endogenous antioxidant enzymes in various pathological and disease conditions (Huang et al., 2015; Yu et al., 2019; Zhang et al., 2019). Melatonin has been associated with the activation of various antioxidant systems and thus helps in maintaining a homeostatic environment in the brain. In particular, melatonin activates SOD GSH, Sirts, and Nrf2/HO-1 pathway. Furthermore, it has been indicated that melatonin plays a vital role in an energy crisis and cellular processes associated with cell survival by activating Sirt1, Sirt3, Sirt6, and other antioxidant pathways and mediators (Ramos et al., 2017). Studies have demonstrated that the antioxidant effects of naturally derived polyphenolic substances involve the activation of the Sirt1-Nrf2 pathway. Similarly, melatonin which acts as a potent antioxidant and endogenous neuroprotective neurohormone also activate the Sirt1-Nrf2 pathway (Hirota et al., 1999; Da Cunha and Arruda, 2017). The Nrf2 is a key endogenous protein and its antioxidant activity is shielded by Keap1 in the cytoplasm. Once stress stimuli trigger Nrf2 activation, its nuclear translocation activates downstream antioxidant machinery including HO-1 and SOD (Jung and Kwak, 2010). In ischemic injury, the activation of this antioxidant machinery starts promptly and continues for 24 h (Yang et al., 2009). Previous studies showed that melatonin activates this machinery in the ischemic model, and thus counteracts the deleterious ischemic effects of oxidative stress (Li and Liu, 2019). In the current study, we did not find a significant overexpression in Nrf2 and HO-1, which could partially be attributed to methodological differences. Consistent studies reiterated the antioxidant potential of melatonin by upregulating the endogenous Nrf2 machinery. A study by Ali et al. showed elevated expression of Nrf2 by melatonin in a neurodegenerative model (Ali et al., 2018). Similarly, Parada et al. demonstrated the upregulation of Nrf2 and HO-1 in the ischemic stroke model (Parada et al., 2014). In our study, we did not notice the overexpression of Nrf2 and its downstream target HO-1. This discrepancy in results may be possibly attributed to species or methodology difference as we used the MCAO rat model, unlikely to photothrombotic mice stroke model by Parada et al. These variations need further exploration to fully understand the potential mechanism of melatonin as a key antioxidant in ischemic conditions. Interestingly, we have found that melatonin stimulates Trx, which has also a key role in the amelioration of oxidative stress associated with ROS and stress kinases, consequently preventing apoptosis and excitotoxicity in ischemic brain injury. Many studies provide evidence for the cross-talk among the endogenous antioxidants and inflammatory mediators, in particular, NF-κB pathways (Schenk et al., 1994). Trx is activated in impaired homeostasis and stress-associated condition and it prevents the neuroinflammation *via* the inhibition of NF-κB nuclear translocation (Schenk et al., 1994). Similarly, our double immunofluorescence and western blot results in this study showed that oxidative stress of ischemic injury (as shown by down expression of Trx) as coupled to the enhanced release of inflammatory cascades (as demonstrated by nuclear p-NF-κB). Interestingly, melatonin pretreatment enhanced the cytoplasmic expression of Trx and down-regulated NF-κB expression, which supports the previous studies and validate the cross-talk between oxidative stress and neuroinflammation (Li and Liu, 2019). It is worth to mention here that melatonin proved a high therapeutic window as the exogenous melatonin dose is most often very high but still, no toxicities and addictive properties have demonstrated with this (Sugden, 1983; Guardiola-Lemaître, 1997), possibly due to its short half-life (20 to 60 min), with a large hepatic first-pass effect and a biphasic elimination pattern (Lane and Moss, 1985). Moreover, the exogenous administration of melatonin elevates both brain Bcl-2 and BDNF levels.

## Conclusions

Our data demonstrated that pretreatment with the endogenous antioxidant and circadian rhythm regulator melatonin rescued the post-MCAO-detrimental outcomes such as oxidative stress-associated MAP kinases, neuroinflammation, and neurodegeneration. Thus, considering our findings, we believe that supplementation with the endogenous antioxidant neurohormone would attenuate the detrimental outcomes of MCAO such as oxidative stress-associated MAPK p-P38/p-JNK, neuroinflammation, and neurodegeneration. The present study revealed the innate immune activation as demonstrated by TNF-α/IL-1 and glial activation, accompanied by oxidative stress signaling (NF-κB/COX2/iNOS) and enhanced anti-oxidative systems such as Nrf2/HO-1/Trx. More interestingly, both systems may work towards the JNK/P38 systems. It is worth mention that our parallel projects screening natural and synthetic compounds targeting JNK/P38 *via* molecular docking also identified melatonin as a potential binding ligand. Moreover, we are also working on the different formulation of melatonin based nanoparticles in ischemic brain injury. We will continue further studies on melatonin and its formulation in ischemic brain injuries. Thus, future preclinical and clinical investigations may warrant a complete pharmacological profile of melatonin and its formulation along with the lifestyle approach to prevent, rescue, and treat MCAO-associated injuries and morbidities.

## Data Availability Statement

The raw data supporting the conclusions of this article will be made available by the authors, without undue reservation, to any qualified researcher.

## Ethics Statement

The animal study was reviewed and approved by Ethical Committee Peking University. Written informed consent was obtained from the owners for the participation of their animals in this study.

## Author Contributions

Conceptualization: LL and FS. Methodology: FS, PK, and TA. Resources: LL, ZT, and SL. Writing: LL, FS, AA, and RA. Review and editing: FS, AA, RA, and TA. Co-supervision: TA. Funding acquisition: LL and SL. Supervision: SL and FS. All authors contributed to the article and approved the submitted version.

## Funding

This research work is supported by the research foundation of Huazhong University of Science and Technology Union Shenzhen Hospital No: F202006281838, and Natural Science Foundation of Shenzhen University General Hospital No: SUGH2020QD015.

## Conflict of Interest

The authors declare that the research was conducted in the absence of any commercial or financial relationships that could be construed as a potential conflict of interest.
